# Robust peptide/RNA complexes prepared with microfluidic mixing for pulmonary delivery by nebulisation

**DOI:** 10.1007/s13346-024-01773-w

**Published:** 2025-01-18

**Authors:** Cheng Ma, Michael Y. T. Chow, Chengyang Zhang, Paulina Goldbaum, Jamie Chien-Ming Hsieh, Jenny K. W. Lam

**Affiliations:** 1 UCL School of Pharmacy, University College London, 29-39 Brunswick Square, London, WC1N 1AX UK; 2 Department of Pharmacology and Pharmacy, LKS Faculty of Medicine, The University of Hong Kong, 21 Sassoon Road, Pokfulam, SAR Hong Kong; 3 Advanced Biomedical Instrumentation Centre, Hong Kong Science Park, New Territories, Shatin, SAR Hong Kong; 4 Medway School of Pharmacy, Central Avenue, University of Kent, Chatham Maritime, Canterbury, ME4 4TB UK

**Keywords:** Aerosolisation, Inhalation, mRNA, siRNA, Transfection, Vibrating mesh nebuliser

## Abstract

Small interfering RNA (siRNA) and messenger RNA (mRNA) have drawn considerable attention in recent years due to their ability to modulate the expression of specific disease-related proteins. However, it is difficult to find safe, robust, and effective RNA delivery systems suitable for pulmonary delivery to treat lung diseases. In this study, two cationic peptides, namely LAH4-L1 and PEG_12_KL4, were employed as non-viral vectors for siRNA and mRNA delivery. Four formulations (i.e. LAH4-L1/siRNA; PEG_12_KL4/siRNA; LAH4-L1/mRNA and PEG_12_KL4/mRNA) were investigated. Microfluidic mixing method was utilised to fabricate RNA complexes in a controllable and reproducible manner. Upon optimisation of the microfluidic mixing protocol, a vibrating mesh nebuliser was employed to aerosolise the RNA complexes, and their transfection efficiency was evaluated on A549 and BEAS-2B cells. Following nebulisation, inhalable mist was generated for all RNA formulations with mass median aerodynamic diameter below 5 μm. Although the hydrodynamic particle sizes of the RNA complexes were significantly reduced to around 100 nm after nebulisation regardless of the original size of the complexes prior to nebulisation, the RNA binding efficiency and the in vitro RNA transfection ability of all the peptide formulations were successfully preserved with no significant differences compared to the same system before nebulisation. The current result indicates that both LAH4-L1 and PEG_12_KL4 hold significant potential for future clinical application for pulmonary siRNA and mRNA delivery through nebulisation.

**Graphical Abstract:**

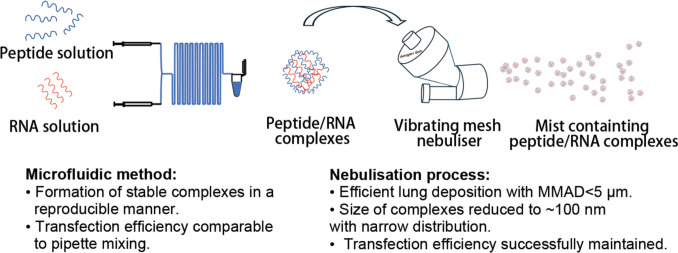

**Supplementary Information:**

The online version contains supplementary material available at 10.1007/s13346-024-01773-w.

## Introduction

Small interfering RNA (siRNA) and messenger RNA (mRNA) have emerged as an important class of therapeutics in recent years. The former inhibits specific gene expression through RNA interference whereas the latter instructs the cells to make specific proteins to produce therapeutic effects. Both RNAs have huge potential for treating a wide range of lung diseases such as severe asthma, chronic obstructive pulmonary disease, respiratory viral infections, lung cancer, and cystic fibrosis [1–5]. The major hurdle to their clinical applications of treating lung diseases is the lack of efficient RNA delivery system to the lung [6–8].

Delivery of RNA through the pulmonary route can maximise the concentration at the site of action in the airways for rapid local effect and reduce the RNA dose required as well as systemic exposure and side effects [6, 9]. Dry powder inhaler (DPI) and nebuliser are the two most commonly investigated inhaler devices to deliver RNAs to the lung. DPI formulation has the advantages of providing a good stability of RNA in a solid dosage form that extends the shelf-life and avoids cold-chain logistics. DPI is also portable and relatively simple to use. However, most DPIs are breath-actuated, which creates problem for some patients whose lung functions are incapable of generating sufficient inspiratory effort to disperse the powder properly, leading to poor lung deposition and treatment efficacy [10, 11]. On the other hand, nebulisation is less convenient than DPI as nebuliser device is generally bulkier, requires electricity for operation and routine maintenance, and the liquid formulation is less stable compared to its solid counterpart. However, nebulisation allows patients to inhale through tidal breathing for drug administration and lung deposition, making it more suitable for a broad range of patients, particularly for those who are critically ill or have severely compromised lung function or even unconscious patients [12].

siRNA and mRNA are negatively charged hydrophilic macromolecules that are susceptible to degradation. Both require a delivery vector to promote cellular uptake and offer protection to avoid premature RNA degradation. Lipid nanoparticles (LNPs) are the most popular non-viral vectors being examined to date for this role. Despite their success in mRNA vaccines and therapeutic siRNA products [13, 14], their application has yet to reach beyond parenteral administration. Depending on the inhalation method, the drying process during powder production (e.g. spray drying) or the nebulisation process during drug administration may introduce thermal, shear and/or interfacial stresses, leading to alteration of the structural integrity of LNPs, affecting the transfection efficiency of the delivery system [15]. More importantly, the airway lining fluid is rich in phospholipids and pulmonary surfactants which may easily destabilise the LNPs [16], resulting in the loss of structural features of LNPs and hence impairing the transfection efficiency. Alternatively, synthetic peptides have emerged to be promising non-viral vectors for RNA delivery [17]. These peptides can be used to functionalise LNPs and polymeric nanoparticles to achieve specific cellular uptake and improve intracellular trafficking, or as delivery vectors on their own [18, 19]. The latter are usually cationic to allow electrostatic interaction with the negatively charged RNA to form nanosized complexes. Peptide-based RNA vectors have attracted significant interest due to their low cost, simple preparation, tuneable sizes and large-scale production. There have been some successes of using peptides for delivering siRNA and mRNA into the lungs of animals [20–22].

In this study, two synthetic amphipathic peptides, namely LAH4-L1 and PEG_12_KL4 were investigated for pulmonary RNA delivery by nebulisation. LAH4-L1 is a pH responsive peptide that can promote endosomal escape whereas PEG_12_KL4 is a surfactant peptide. Both peptides were previously demonstrated to be effective in transfecting RNAs in lung epithelial cells [20, 23, 24]. On a lab scale production, the peptide/RNA complexes are usually prepared by simple bulk mixing of peptide and RNA at appropriate ratio with a pipette manually. For pilot or industrial scale production, a reliable and scalable method that can generate uniform nanosized complexes in a reproducible manner is critical. Microfluidic approach has been successfully applied to the production of LNPs [25]. It can enhance the mixing efficiency and allows continuous nanoparticle production process. With the use of microfluidic chip devices, the flow rate and hence mixing rate can be controlled precisely. Here, peptide/siRNA and peptide/mRNA complexes were prepared by microfluidic mixing and their particle size distribution, stability and transfection efficiency were compared with those prepared by pipette mixing. The complexes were then aerosolised using a vibrating mesh nebuliser, and their suitability for inhalation was evaluated in terms of aerodynamic particle size distribution, stability, and transfection efficiency.

## Material and methods

### Material

LAH4-L1 and PEG_12_KL4 peptides (Table 1) were synthesised by EZBiolab (NJ, USA). Cy3-labeled siRNA (siGLO Cyclophilin B Control siRNA) was purchased from Horizon Discovery Ltd (Cambridge, UK). Luciferase mRNA (CleanCap® FLuc mRNA, 1929 nucleotides) was purchased from TriLink BioTechnologies (CA, USA). Cy5-labeled mRNA (ARCA Cy5 EGFP mRNA) was purchased from ApexBio Technology (TX, US). Dulbecco’s modified eagle medium (DMEM), keratinocyte serum-free medium (SFM, combined with bovine pituitary extract and human recombinant epidermal growth factor), OptiMEM, foetal bovine serum (FBS), Antibiotic–Antimycotic (100x, penicillin, streptomycin, and amphotericin B), Lipofectamine 2000 transfection reagent, glyceraldehyde 3-phosphate dehydrogenase (GAPDH) siRNA (21 base pairs) and scramble siRNA were purchased from ThermoFisher (MA, USA). QuantiFluor ® RNA system, report lysis 5 × buffer, and luciferase assay system were obtained from Promega Corporation (Southampton, UK). Ultrapure water was obtained from the Purelab Chorus system (ELGA Labwater, High Wycombe, UK).

**Table 1:** Sequence, molecular weight (MW) and net charge (at pH 7) of peptides used in this study. PEG_12_ is a monodisperse PEG with 12 monomers

Peptide	Sequence	MW	Net Charge
LAH4-L1	KKALLAHALHLLALLALHLAHALKKA-NH_2_	2780	+ 4
PEG_12_KL4	PEG_12_-KLLLLKLLLLKLLLLKLLLLK-NH_2_	3068	+ 5

### Preparation of peptide/RNA complexes

All four peptide/RNA complexes (i.e. LAH4-L1/siRNA; LAH4-L1/mRNA; PEG_12_KL4/siRNA; and PEG_2_KL4/mRNA) were prepared at a ratio of 10:1 (w/w) using microfluidic mixing and pipette mixing method. GAPDH siRNA and luciferase mRNA were used unless stated otherwise. The microfluidic mixing method was first optimised (Table S1 and S2, supplementary information). In the optimised protocol, peptide solution (1.4 mg/mL) and RNA solution (0.14 mg/mL) were first prepared in ultrapure water. The two solutions were injected separately into the two inlets of a microfluidic chip (hydrophilised PC Fluid 186, Microfluidic ChipShop, Jena, Germany) at a flow rate of 3 μL/min controlled by a syringe pump (KDS Legato™ 210 syringe pump, KD Scientific, MA, USA). The first 20 μL of the mixed solution from the microfluidic chip outlet was discarded and the rest was collected. With the pipette mixing method, the peptide solution (1.4 mg/mL) was added to the RNA solution (0.14 mg/mL) at equal volume. The solutions were manually mixed by pipetting for 10 s followed by brief vortexing (for siRNA complexes) or manual inverting (for mRNA complexes) for 10 s. The mixtures were incubated at room temperature for at least 30 min. In both preparation methods, peptide/RNA complexes at RNA concentration of 70 μg/mL were obtained, and they were either further diluted or used directly as prepared.

### Measurement of particle size distribution and zeta potential

Particle size distribution and zeta potential of peptide/RNA complexes was measured by dynamic light scattering and electrophoretic light scattering, respectively (Zetasizer nano ZS, Malvern Panalytical, Malvern, UK). For size measurement, the samples were measured at various time points after complexes formation and after nebulisation. The sample was added to a disposable microcuvette for measurement. The data were presented as mean hydrodynamic diameter, polydispersity index (PDI). For zeta potential measurement, the complexes were first diluted in 10% PBS to achieve RNA concentration of 14 μg/mL. The samples were then loaded into a capillary zeta cell for measurement.

### Morphology study using transmission electron microscope (TEM)

TEM was used to examine the morphology of peptide/RNA complexes. Discharged copper grids were immersed in the solutions of complexes (at RNA concentration of 70 μg/mL) for 1 min. The grids were then stained with 4% (w/v) uranyl acetate for 20 s followed by washing with distilled water. Excess liquids were removed by filter paper and the examples were air-dried. The samples were viewed under TEM (FEI Tecnai G2 20 S-TWIN, FEI Company, USA), and images were taken at 9900 and 19500 folds of magnification with a Gatan ORIUS SC600 Model 831 CCD Camera (2.7 k × 2.7 k pixel) with Digtalmicrograph software (Gatan, Inc., CA, US).

### Nebulisation

A vibrating mesh nebuliser, Aerogen Solo (Aerogen, Galway, Ireland), was employed in this study to nebulise the peptide/RNA complexes. The complexes prepared were either diluted in ultrapure water prior to nebulisation to obtain a final RNA concentration of 14 μg/mL (for particle size measurement and in vitro transfection) or use directly as prepared (for cascade impactor study). The samples were loaded into the medication cup of Aerogen Solo. For particle size and transfection studies, a centrifuge tube was connected to the output of the nebuliser to collect the aerosol. The samples were centrifuged at 2,000 rpm for 10 min to obtain the solution adhered to the wall of the tube. The collected sample was transferred to a microcentrifuge tube for further evaluations.

### RNA binding

To measure the RNA binding ability of peptides, fluorescent dye (QuantiFluor® RNA system) was employed to detect free and total RNA in the system before and after nebulisation. The working dye solution and standard curves (in the presence and absence of SDS) were prepared according to the manufacturer protocol. The dye solution was added to the peptide/RNA complexes to quantify the free unbound RNA. SDS (10 mM) was added to the complexes to release the bound RNA, and the dye solution was added to quantify the total RNA in the system. The fluorescence intensity was measured by GloMax Explorer according to the programme protocol (Promega, Southampton, UK) at λ_ex_ 475 nm and λ_em_ 500–550 nm. The bound RNA was calculated by subtracting the free RNA from the total RNA. The RNA binding was calculated as the percentage of bound RNA in total RNA in the sample.

### Cell culture

A549 cells (human alveolar epithelial adenocarcinoma), and BEAS-2B cells (human bronchial epithelial cells) were obtained from ATCC (Manassas, VA, USA). A549 cells were cultured in DMEM supplemented with 10% FBS and antibiotic–antimycotic. BEAS-2B cells were cultured in Keratinocyte SFM supplemented with bovine pituitary extract, human recombinant epidermal growth factor, and antibiotic–antimycotic. The cells were sub-cultured twice a week and were maintained in a humified incubator at 37˚C and 5% CO_2_.

### In vitro siRNA transfection

A549 and BEAS-2B cells were seeded on 6-well plates at a density of 1.6 × 10^5^ cells/well and 2 × 10^5^ cells/well, respectively, 24 h before transfection. The peptide/siRNA complexes prepared with GAPDH siRNA or scramble siRNA were diluted in serum-free OptiMEM to obtain the final volume of 1 mL per well at siRNA concentrations of 50, 25, and 12.5 nM (i.e. 0.67, 0.33, and 0.17 μg/mL, respectively). At 5 h post-transfection, the transfection media were removed and replaced with normal culture media. At 48 h post-transfection, the cells were washed and lysed. The total protein concentrations of cell lysates were measured by Bradford assay. The GAPDH level within the cell lysates was quantified with the Western blotting assay as previously described [24]. The density of GAPDH protein bands was normalised with β-actin bands, and the siRNA transfection efficiency was expressed as the percentage of the remaining expression of GAPDH protein bands in the peptide/GAPDH siRNA treated groups compared to the GAPDH protein bands of the corresponding peptide/scramble siRNA treated groups.

### In vitro mRNA transfection

A549 and BEAS-2B cells were seeded on 24-well plates at a density of 5 × 10^4^ cells/well and 1 × 10^5^ cells/well, respectively, 24 h before transfection. The peptide/mRNA complexes prepared were diluted in serum-free OptiMEM to achieve the final mRNA concentration of 1, 0.5, and 0.25 µg/well (0.5 mL of sample per well). At 5 h post-transfection, the transfection media were removed and replaced with normal culture media. At 24 h post-transfection, the cells were washed and lysed. The total protein contents in cell lysates were determined by Bradford assay. The luciferase activities were measured as previously described [20]. The luminescence was measured with GloMax Explorer (Promega, Southampton, UK). The relative luminescence unit (RLU) was calculated based on the luminescence unit per μg of total protein.

### Cascade impactor study

Next Generation Impactor (NGI) (Copley Scientific Limited, Nottingham, UK) was employed to evaluate the aerosol profile of nebulised peptide/RNA complexes. Prior to the experiment, NGI was pre-cooled at 5˚C for at least 90 min, and it was placed on an ice bath during evaluation. The Aerogen Solo was fit on an Aerogen Ultra chamber which was connected to the mouth of the induction port of NGI with a rubber adaptor. A vacuum pump (HCP5 high-capacity pump, Copley Scientific Limited, Nottingham, UK) was connected to the NGI to provide a constant flow rate of 15.0 ± 0.15 L/min during the experiment. An external filter (PARI Medical Ltd, Surrey, UK) was placed between the NGI outlet and the pump. The complexes were prepared with Cy3-labeled siRNA or Cy5-labeled mRNA, and the samples were nebulised through the Aerogen Solo. After nebulisation was completed (500 µL of peptide/siRNA complexes and 800 µL of peptide/mRNA complexes), the NGI was rested on ice for additional 20 min to allow deposition of the suspended aerosol. Samples deposited at each stage were then collected with PBS and the amount of RNA deposited in each stage was quantified by measuring the fluorescence intensity (at λ_ex_ 530 nm and λ_em_ 565 nm for Cy3-labeled siRNA; at λ_ex_ 640 nm and λ_em_ 670 nm for Cy5-labeled mRNA) using a plate reader (SpectraMax M2, Molecular Device, Berkshire, UK) against a standard curve. The emitted fraction (EF) was calculated as the percentage of the emitted dose (from induction port to micro-orifice collector, MOC) in the total recovered dose. Fine particle fraction (FPF) was calculated as percentage of particles with aerodynamic size < 5 µm relative to the emitted dose. Mass median aerodynamic diameter (MMAD) was defined as the aerodynamic diameter at which half of the particles by mass are larger and the other half smaller.

### Statistical analysis

The grouped data were analysed using two-way ANOVA followed by post-hoc analysis with Šidák analysis for multiple group comparisons with each other and Dunnett for comparisons with one control group. All statistical analyses were performed on GraphPad Prism Version 9.5.0. All experiments were repeated at least three times independently unless stated otherwise.

## Results

### Physicochemical properties peptide/RNA complexes prepared by the two mixing methods

Four different peptide/RNA systems were investigated here, including two peptides (LAH4-L1 and PEG_12_KL4) and two types of RNA (siRNA and mRNA). The complexes of all four systems were prepared at 10:1 peptide to RNA ratio (w/w) that showed the best performance according to previous studies [20, 23, 24]. This ratio is equivalent to the N/P ratios of 4.5:1, 5.1:1, 4.7:1, and 5.3:1 for LAH4-L1/siRNA, PEG_12_KL4/siRNA, LAH4-L1/mRNA, and PEG_12_KL4/mRNA, respectively. First, the preparation of peptide/RNA complexes using microfluidic mixing method was optimised by examining the effect of the total output flow rate and the input flow rate ratio of peptide to siRNA inlets. It was found that a low total output flow rate of 6 μL/min and the input flow ratio of 1:1 (i.e. 3 μL/min for each inlet) could achieve the smallest and most stable complexes for all four systems (Fig. S1 and S2 supplementary information), and this microfluidic mixing protocol was adopted for subsequent studies. The complexes prepared by microfluidic mixing and pipette mixing approaches in this study were then compared side by side.

**Fig. 1:**
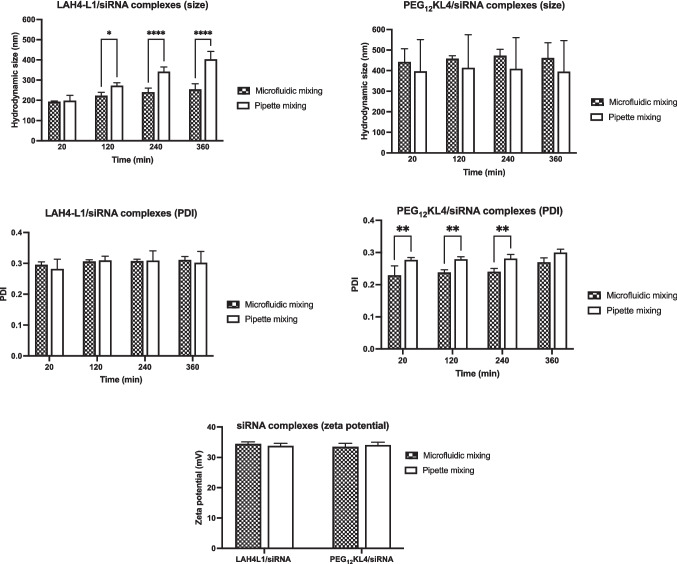
Particle size distribution and zeta potential of peptide/siRNA complexes. LAH4-L1/siRNA and PEG_12_KL4/siRNA complexes were prepared at a 10:1 ratio (w/w) at siRNA of 70 μg/mL by microfluidic mixing or pipette mixing. The hydrodynamic size and polydispersity index (PDI) were measured at different time point after complex formation. For zeta potential measurement, the complexes were diluted in 10% PBS to achieve siRNA concentration of 14 μg/mL. The data were presented as mean ± standard deviation (n = 3–4). The data were analysed by two-way ANOVA followed by Šidák’s multiple comparisons test. *p < 0.05, **p < 0.01, ****p < 0.001

For siRNA (Fig. 1), the microfluidic mixing generally produced more stable LAH4-L1/siRNA complexes. The complexes prepared with pipette mixing grew in size from just below 200 nm to over 300 nm after 6 h of preparation, and they became significantly larger than their microfluidic counterpart. For PEG_12_KL4/siRNA complexes, they were larger in size of around 400 nm. There was no significant difference between the two preparation methods. However, the size of PEG_12_KL4/siRNA complexes prepared with microfluidic mixing had a smaller standard deviation and exhibited a lower PDI compared to pipette mixing, with a significant difference observed at 20, 120, and 240 min after preparation, implying that the microfluidic mixing method was more consistent and capable of producing siRNA complexes with narrower size distribution. Both LAH4-L1/siRNA and PEG_12_KL4/siRNA complexes exhibited a zeta potential of around + 35 mV, and there was no significant difference between pipette and microfluidic mixing.

**Fig. 2:**
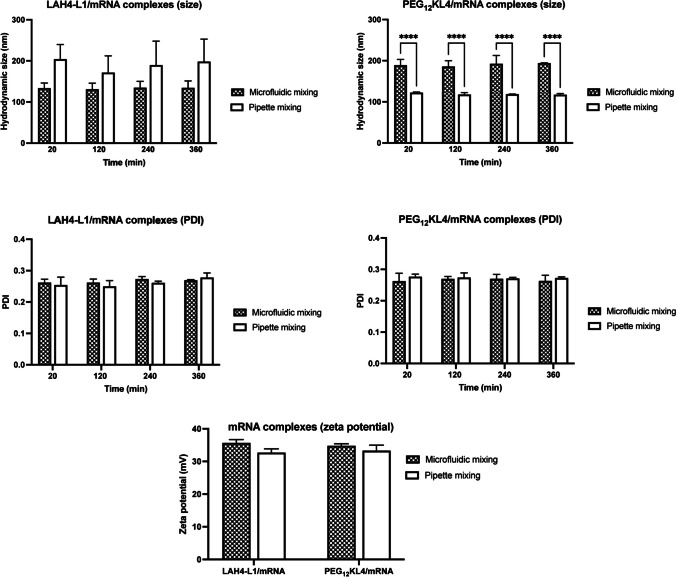
Particle size distribution and zeta potential of peptide/mRNA complexes. LAH4-L1/mRNA and PEG_12_KL4/mRNA complexes were prepared at a 10:1 ratio (w/w) at mRNA of 70 μg/mL by microfluidic mixing or pipette mixing. The hydrodynamic size and polydispersity index (PDI) were measured at different time point after complex formation. For zeta potential measurement, the complexes were diluted in 10% PBS to achieve mRNA concentration of 14 μg/mL. The data were presented as mean ± standard deviation (n = 3–4). The data were analysed by two-way ANOVA followed by Šidák’s multiple comparisons test. ****p < 0.001

For mRNA (Fig. 2), the LAH4-L1/mRNA complexes produced with microfluidic mixing tended to have a smaller particle size of below 150 nm, whereas those prepared with pipette mixing exhibited size approaching 200 nm. However, there was no statistically significant differences between the two. On the contrary, the PEG_12_KL4/mRNA complexes prepared by microfluidic mixing had a significantly larger size of around 200 nm, compared to those prepared by pipette mixing with size of just above 100 nm. There were no differences in terms of PDI of the complexes prepared with both methods for either peptide. Similar to siRNA complexes, the zeta potential of all mRNA complexes was around + 35 mV with no significant difference between the two mixing methods.

**Fig. 3:**
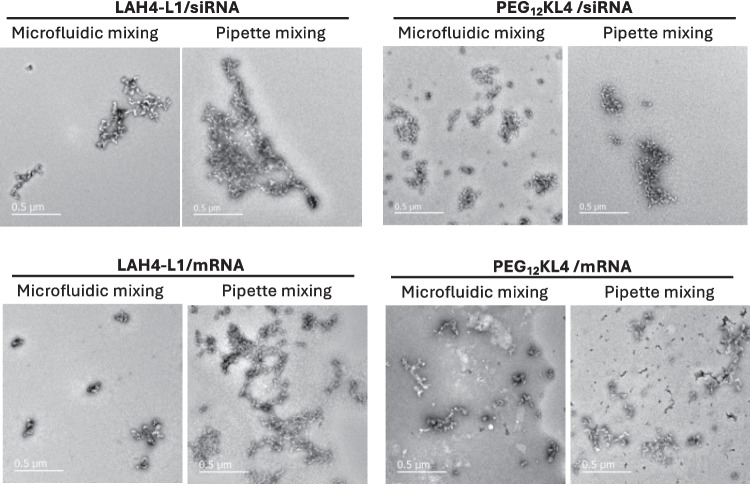
Transmission electron microscopy (TEM) images of peptide/RNA complexes. The complexes were prepared at 10:1 peptide/RNA ratio (w/w) at RNA concentration of 70 μg/mL. The samples were stained with 4% uranyl acetate prior to imaging. Scale bar = 500 nm

The complexes were also observed under TEM (Fig. 3). All of them showed sign of aggregation but to a different extent, possibly due to the relatively high concentration of complexes used for imaging. In general, complexes formed with pipette mixing tend to have a higher level of aggregation, as seen with LAH4-L1/siRNA, PEG_12_KL4/siRNA, and LAH4-L1/mRNA complexes. The PEG_12_KL4/mRNA complexes formed by microfluidic mixing and pipette mixing however did not appear to have any noticeable differences.

### Transfection efficiency of the RNA complexes prepared with the two mixing methods

**Fig. 4:**
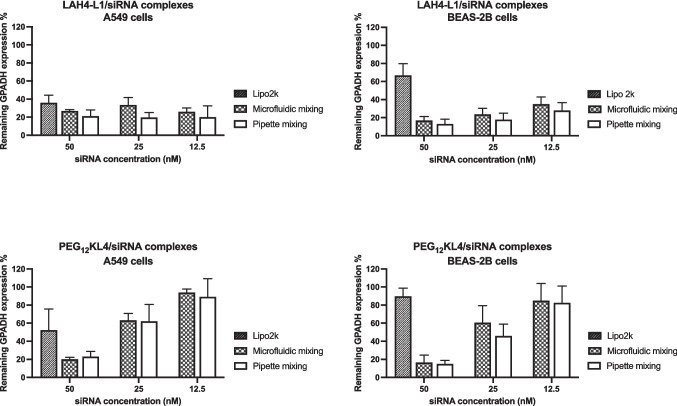
Transfection of peptide/siRNA complexes on A549 and BEAS-2B cells. LAH4-L1/siRNA and PEG_12_KL4/siRNA complexes were prepared at a 10:1 ratio (w/w) by microfluidic mixing or pipette mixing. siRNA targeting GAPDH was used. The cells were also transfected with Lipofectamine 2000 (Lipo2k) at a siRNA concentration of 50 nM as control. The % of remaining GAPDH was measured at 48 h post-transfection by Western blot analysis and the densitometry results were presented as mean ± standard deviation (n = 3). The data were analysed by two-way ANOVA followed by Šidák’s multiple comparisons test. No significant difference was observed in siRNA transfection efficiency between complexes prepared by microfluidic mixing and pipette mixing

**Fig. 5:**
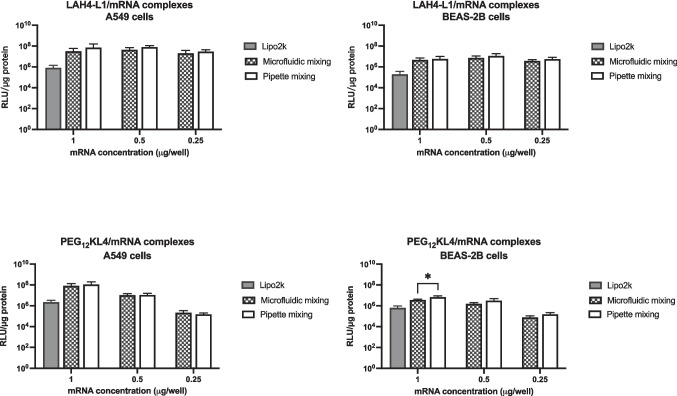
Transfection of peptide/mRNA on A549 and BEAS-2B cells. LAH4-L1/mRNA and PEG_12_KL4/mRNA complexes were prepared at a 10:1 ratio (w/w) by microfluidic mixing or pipette mixing. mRNA encoding luciferase was used. The cells were also transfected with Lipofectamine 2000 (Lipo2k) at a mRNA dose of 1 μg per well as control. The relative light unit (RLU) per μg protein was measured at 24 h post-transfection. The results were presented as mean ± standard deviation (n = 3). The data were analysed by two-way ANOVA followed by Šidák’s multiple comparisons test. *p < 0.05

The transfection efficiencies of siRNA complexes (Fig. 4) and mRNA complexes (Fig. 5) prepared by the two mixing methods were compared at three different RNA concentrations on A549 and BEAS-2B cell lines, both of which are human lung epithelial cells. Representative Western blot images of cells transfected with siRNA were also shown (Fig. S3, supplementary information). In general, the two methods were comparable in preparing complexes with similar RNA transfection efficiency. Significant difference between the two was observed with the transfection of PEG_12_KL4/mRNA complexes on BEAS-2B cells at 1 μg/well of mRNA. In this case, the pipette mixing method achieved a better transfection efficiency, but the difference was not substantial. For all other conditions, there were no significant differences between the two preparation methods.

### Effect of nebulisation on particle size distribution, RNA binding and transfection efficiency

**Fig. 6:**
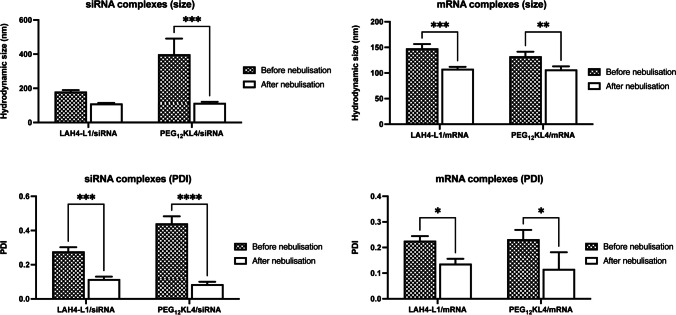
Particle size distribution of peptide/RNA complexes before and after nebulisation. All the peptide/RNA complexes were prepared at a 10:1 ratio (w/w) at RNA concentration of 14 μg/mL by microfluidic mixing. The hydrodynamic size and polydispersity index (PDI) were measured by dynamic light scattering. The data were presented as mean ± standard deviation (n = 3–4). The data were analysed by two-way ANOVA followed by Šidák’s multiple comparisons test. *p < 0.05, **p < 0.01. ***p < 0.005 and ****p < 0.001

**Fig. 7:**
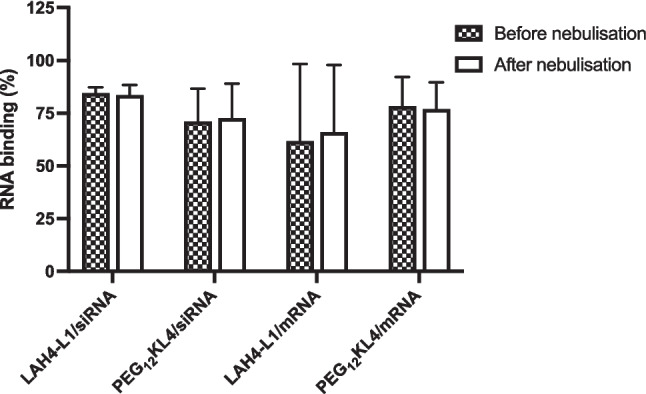
RNA binding of peptide before and after nebulisation. All peptide/RNA complexes were prepared at 10:1 ratio (w/w) by microfluidic mixing. The data were presented as mean ± standard deviation (n = 3). No significant difference in siRNA transfection efficiency was observed before and after nebulisation

**Fig. 8:**
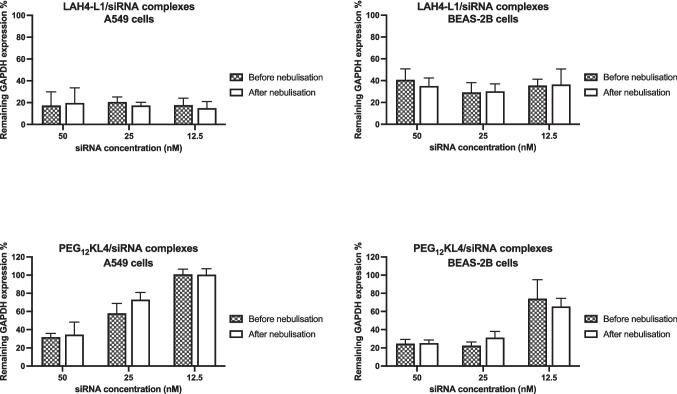
Transfection of peptide/siRNA complexes before and after nebulisation on A549 and BEAS-2B cells. LAH4-L1/siRNA and PEG_12_KL4/siRNA complexes were prepared at a 10:1 ratio (w/w) by microfluidic mixing. siRNA targeting GAPDH was used. The % of remaining GAPDH was measured at 48 h post-transfection by Western blot analysis and the densitometry results were presented as mean ± standard deviation (n = 3). The data were analysed by two-way ANOVA followed by Šidák’s multiple comparisons test. No significant difference in siRNA transfection efficiency was observed before and after nebulisation

**Fig. 9:**
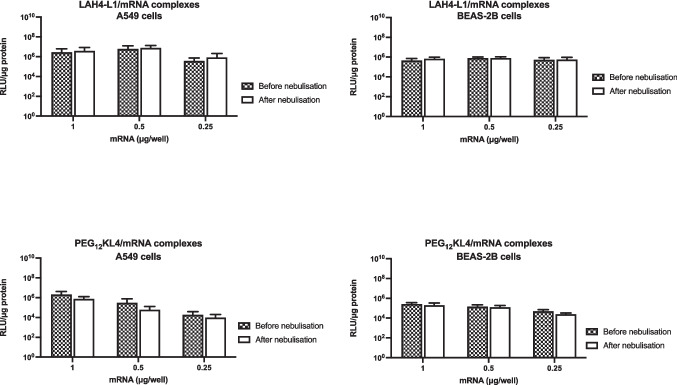
Transfection of peptide/mRNA complexes before and after nebulisation on A549 and BEAS-2B cells. LAH4-L1/mRNA and PEG_12_KL4/mRNA complexes were prepared at a 10:1 ratio (w/w) by microfluidic mixing. siRNA targeting GAPDH was used. mRNA encoding luciferase was used. The relative light unit (RLU) per μg protein was measured at 24 h post-transfection. The results were presented as mean ± standard deviation (n = 3). The data were analysed by two-way ANOVA followed by Šidák’s multiple comparisons test. No significant difference in mRNA transfection efficiency was observed before and after nebulisation

With the similar biological activities between RNA complexes formed by microfluidic mixing and pipette mixing, only the former method was used in the nebulisation studies. During the nebulisation process, the shear and interfacial stresses may impact on the stability of biological molecules. Previous studies showed that nebulisation could promote aggregation of proteins [26]. Here, the particle size of the RNA complexes was compared before and after nebulisation (Fig. 6). Apart from LAH4-L1/siRNA complexes, all other formulations experienced a significant reduction of particle size after nebulisation. Moreover, all complexes after nebulisation exhibited particle size of around 100 nm regardless of their size prior to nebulisation. The most prominent effect was observed with PEG_12_KL4/siRNA complexes in which the hydrodynamic size of particles reduced from around 400 nm before nebulisation to below 120 nm after nebulisation. They also displayed a narrower size distribution, with PDI value closed to 0.1 in all formulations after nebulisation. It appeared that the nebulisation process could de-aggregate the RNA complexes. The extent of RNA binding with the peptides was examined by fluorescence assay (Fig. 7). LAH4-L1 exhibited a higher binding affinity for siRNA with around 85% of siRNA bound to the peptide. In contrast, just over 60% of mRNA bound to the LAH4-L1. On the other hand. PEG_12_KL4 displayed similar binding affinity for siRNA and mRNA, with RNA binding in the range of 70 to 80%. Despite the considerable changes in particle size of peptide/RNA complexes following nebulisation, there was no substantial change in RNA binding efficiency. Similarly, the transfection efficiency of the RNA complexes was not affected by the nebulisation process. Both LAH4-L1 and PEG_12_KL4 showed similar level of siRNA transfection (Fig. 8 and Fig. S4, supplementary information) or mRNA transfection (Fig. 9) on the two tested cell lines before and after nebulisation, with no significant differences between them.

### Aerosol performance evaluation using NGI

**Fig. 10:**
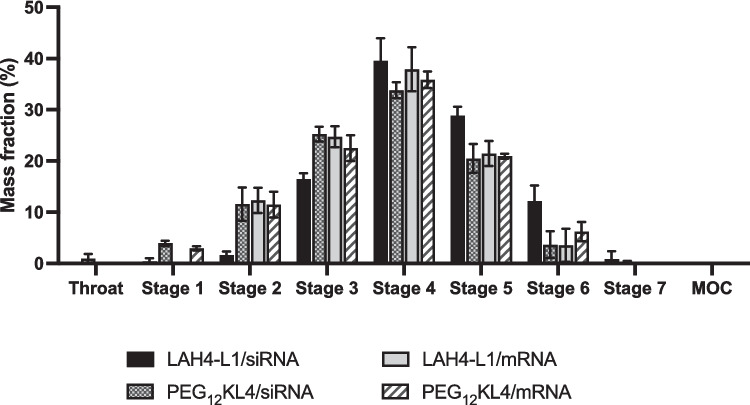
Deposition profile of peptide/RNA complexes in Next Generation Impactor (NGI) following nebulisation. The NGI was operated at a flow rate of 15 L/ min. All complexes were prepared at a peptide to RNA ratio of 10:1 (w/w) by microfluidic mixing. The deposition of across the throat, stage 1–7 and micro-orifice collector (MOC) were calculated as mass fraction (%) of the emitted dose. The stage cut-off diameters were 14.1 µm, 8.61 µm, 5.39 µm, 3.30 µm, 2.08 µm, 1.36 µm, and 0.98 µm for stage 1–7, respectively. The results were presented as mean ± standard deviation (n = 3)

All four peptide/RNA complexes showed similar deposition profile in NGI following nebulisation by Aerogen Solo, which is a vibrating mesh nebuliser (Fig. 10). An EF and FPF of over 90% and 50%, respectively, were achieved by all four formulations (Table 2), with MMAD ranged between 3 and 5 μm. Overall, the NGI results indicated that the aerosols generated by the nebuliser were generally suitable for inhalation.

**Table 2:** Aerodynamic properties of the nebulised peptide/RNA complexes evaluated by the Next Generation Impactor (NGI). The emitted fraction (EF), fine particle fraction (FPF) and mass median aerodynamic diameter (MMAD) were presented as mean ± standard deviation (n = 3)

	EF (%)	FPF (%)	MMAD (µm)
LAH4-L1/siRNA	99.0 ± 1.76	77.0 ± 1.05	3.58 ± 0.180
PEG_12_KL4/siRNA	94.5 ± 1.47	52.6 ± 6.32	4.85 ± 0.386
LAH4-L1/mRNA	91.1 ± 11.5	56.4 ± 2.10	4.63 ± 0.130
PEG_12_KL4/mRNA	94.5 ± 1.93	57.0 ± 2.85	4.56 ± 0.176

## Discussion

With the success of Onpattro, an LNP formulation of siRNA used for the treatment of polyneuropathy caused by the hereditary transthyretin-mediated amyloidosis [27], and the mRNA vaccines developed by Moderna and Pfizer, both of which employ LNPs to deliver mRNA vaccine against COVID-19 [13], LNPs have overshadowed other RNA delivery systems and dominated this field of research. For pulmonary RNA delivery however, LNPs face additional challenges concerning their stability. Firstly, a number of studies reported that nebulisation led to destruction or agglomeration of LNPs, causing detrimental effect on RNA encapsulation and their transfection efficiency [15, 28, 29]. It could be due to the delicate structure of LNPs in which their functionality depends on. Secondly, the rich contents of phospholipids in the pulmonary surfactant and the mucus layer in the airways may pose a high risk of interacting and destabilising the LNPs [16, 30]. In this regard, our group has focused on developing peptide-based systems which are structurally simpler and physically more robust than LNPs for pulmonary RNA delivery.

Both LAH4-L1 and PEG_12_KL4 peptides used in this study can form nanosized complexes with siRNA and mRNA through electrostatic interaction, and they have shown good RNA transfection efficiency in lung epithelial cells [20, 23, 24, 31]. Moreover, these water-soluble peptides do not require organic solvents such as ethanol, nor pH changes during formulation, providing an additional benefit over LNPs. A reliable, reproducible, and scalable production method needs to be considered in the subsequent stage of development. Conventional bulk mixing approach, frequently accomplished by using a pipette manually in lab scale research, often results in heterogenous products with significant batch-to-batch variations [32]. Here, insights were gained from the success of LNPs in the production perspective by adopting the microfluidic approach to prepare RNA complexes. In fact, the use of microfluidic mixing was reported over a decade ago for the preparation of polymer/DNA complexes [33]. Microfluidic mixing offers the advantages of high controllability, homogeneity and reproducibility, and the opportunities for automation and continuous production process [34]. Our first objective was to identify a microfluidic mixing protocol that enables the production of small and reproducible peptide/RNA complexes. A low total output flow rate and a low input flow rate ratio were found to be critical in preparing small and stable peptide/RNA complexes. As laminar flow was expected in the microchannel, mixing was achieved mainly by diffusion which allows the transfer of molecules through the interfacial area [35]. A lower total flow rate increased the contact time per unit area between the two liquids, and hence improved mixing results. For the input flow rate ratio, the larger the difference between the input flow rate of the two solutions, the larger the difference in volume between the two, the larger the size of complexes. This could be explained by the longer diffusion distance between the two solutions, resulting in insufficient mixing. These observations were opposite to the preparation of LNPs where high flow rate tended to yield better mixing and smaller LNPs [25, 36]. As the preparation of LNPs involved the mixing of organic and aqueous phases, the solubility of lipids changed gradually during the mixing process, hence the impact of flow rate and flow rate ratio would be different to the diffusion mixing in our system in which both RNA and peptide are dissolved in aqueous phase.

The microfluidic mixing with our established protocol was then compared with the pipette mixing method. In general, siRNA complexes were larger than mRNA complexes. The short double-stranded siRNA has a relatively long persistence length that renders it a more rigid structure [37], leading to the formation of larger complexes when it binds to the peptides. On the other hand, mRNA is a long single-stranded molecule that is more flexible to allow the formation of smaller and more compact complexes. Microfluidics improved the mixing between LAH4-L1 and siRNA, as seen with the smaller size of complexes with better stability and higher binding affinity compared to the pipette mixing. The effect was less apparent between PEG_12_KL4 and siRNA as the size of complexes were similarly large for microfluidic and pipette mixing, although the former produced complexes with slightly lower PDI. Both LAH4-L1 and PEG_12_KL4 are cationic peptides, but the positive charges are more evenly distributed in LAH4-L1 along the sequence that allows more intimate interaction with the siRNA. PEG_12_KL4 contains a short chain of neutral PEG at one end which may either hinder its interaction with the rigid siRNA to form looser complexes or the PEG self-aligns on the surfaces of the complexes that give rise to larger complexes. Since the zeta potential of PEG_12_KL4/siRNA complexes was positive with a value similar to that of LAH4-L1 (around + 35 mV), it is more probable that the PEG chain was embedded in the complexes rather than exposed to the surface, resulting in the formation of looser complexes.

For mRNA complexes, microfluidic mixing produced smaller LAH4-L1/mRNA complexes than pipette mixing but the differences between the two preparation methods were not reflected on the PDI. On the contrary, the size of PEG_12_KL4/mRNA complexes were larger when they were prepared with microfluidic mixing. It appeared that the current microfluidic mixing protocol was still suboptimal to achieve effective mixing for this system. Overall, the microfluidics could facilitate the mixing between peptide and RNA to a certain extent, and the complexes appeared to be less aggregated as observed in the TEM images. To elucidate the architecture of the complexes, techniques such as small angle neutron scattering and small angle x-ray scattering which provide detail information such as morphology and core/shell structure of nanosized complexes could be considered for future study [38].

Interestingly, the differences in physicochemical properties of peptide/RNA complexes between the two mixing approaches did not translate to their transfection efficiencies in vitro. Out of the 24 transfection conditions tested (i.e. 2 peptides × 2 RNAs × 2 cell lines × 3 RNA doses), 23 of them showed no significant differences between microfluidic and pipette mixing. Hence, we concluded that both mixing methods were highly comparable in producing complexes that demonstrated similar in vitro biological activities. For the purposes of consistency and scalable manufacturing, the subsequent nebulisation study was conducted using complexes prepared with microfluidic approach only.

Vibrating mesh nebuliser was employed in this study because as it is considered to be gentler compared to other types of nebulisers such as jet and ultrasonic nebulisers. When the peptide/RNA complexes were subjected to nebulisation with a vibrating mesh nebuliser, there was a significant reduction in the particle size as well as the PDI. After nebulisation, all complexes (regardless of the RNA type, peptide and the initial size of complexes prior to nebulisation) exhibited a hydrodynamic diameter of around 100 nm with a narrow distribution of PDI around 0.1. This was rather unexpected as previous studies showed that nebulisation often led to an increase in particle size or rupture of LNPs, resulting in lower transfection efficiencies [39, 40]. Unlike jet and ultrasonic nebulisers which contain baffles to trap the large droplets so that only the smaller droplets are inhaled, vibrating mesh nebuliser utilises a piezoelectric crystal which vibrates at a high frequency, forcing the liquid through the perforated mesh to form fine aerosols for inhalation. The lack of baffle in a vibrating mesh nebuliser reduces shear stress and damage to the LNPs. Nonetheless, LNPs gain mobility and are brought into close contact with each other during nebulisation. This could lead to the agglomeration of LNPs, possibly caused by the rearrangement and fusion of lipids from adjacent particles, resulting in irreversible structural change of LNPs [29]. It is challenging to identify suitable compositions of lipids to form LNPs that can endure the nebulisation process. In contrast, our peptide/RNA complexes not only could resist agglomeration induced by nebulisation, but they were also de-aggregated by the vibrating energy during the process, making them particularly suitable and robust for nebulisation. More importantly, there was no significant difference in transfection efficiency in vitro for most of the tested RNA/complexes before and after nebulisation, implying that de-aggregation did not enhance transfection nor impair the integrity of the RNAs.

Finally, the NGI results clearly suggested that Aerogen Solo could successfully deliver peptide/RNA complexes to the deep lung. A satisfactory aerosolisation profile was observed for all the RNA complexes, with a significant portion of the aerosolised particles lied within the range for respiration. Interestingly, the FPF of LAH4-L1/siRNA complexes were higher than other formulations which was rather unexpected as the aerosol size would be largely dependent on the nebuliser. According to the RNA binding assay, LAH4-L1/siRNA complexes exhibited the highest RNA binding % before and after nebulisation (> 80%), suggesting that there was less free RNA in the solution. LAH4-L1/siRNA formulation may have a lower viscosity compared to the others, leading to the formation of droplets with smaller size and hence higher FPF. Nonetheless, with the encouraging transfection results following nebulisation, this work revealed the promising application of peptide/RNA complexes for pulmonary delivery through nebulisation. The next step of the study is to examine the mucus penetration property of the peptide/RNA systems, evaluate the in vivo transfection following nebulisation in animals which will also give us insights on the stability as well as the safety of the aerosolised RNA formulations in the airways.

## Conclusions

In this study, an optimised microfluidic mixing method was established for producing peptide/RNA complexes in a reproducible manner, and the physicochemical properties and biological activities of complexes were comparable to those prepared with pipette mixing. Despite the particle size of the peptide/RNA complexes was significantly reduced after aerosolisation through vibrating mesh nebuliser, possibly due to de-aggregation of the complexes, their vitro transfection efficiency was successfully preserved, and the size of aerosol was suitable for inhalation. These peptide-based RNA delivery systems offer alternative solution to LNPs to the delivery of RNA therapeutics to the lung. Further evaluation on their performance in animal models and investigation on their potential clinical applications such as the treatment of cystic fibrosis and lung cancer are warranted.

## Supplementary Information

Below is the link to the electronic supplementary material.

Supplementary file1 (DOCX 3211 KB)

## Data Availability

The data generated in this study are available from the corresponding author on reasonable request.
