# Supplementary material for: Robust peptide/RNA complexes prepared with microfluidic mixing for pulmonary delivery by nebulisation
Source: Drug Deliv Transl Res. 2025 Jan 18;15(8):2765–78. doi: 10.1007/s13346-024-01773-w (PMC12213855; doi:10.1007/s13346-024-01773-w)
Supplement: Supplementary file 1 — Supplementary file1 (DOCX 3211 KB) [file 13346_2024_1773_MOESM1_ESM.docx]

**Supplementary Information**

**Table S1.** Factorial designs to optimise the microfluidic mixing method for the preparation of peptide/siRNA complexes. LSM refers to LAH4-L1/siRNA complexes; PSM refers to PEG_12_KL4/siRNA complexes. FRR is flow rate ratio (peptide to siRNA); TF is total flow rate.

|  | FRR | TF  (μL/min) | Inlet 1: peptide solution | | |  | Inlet 2: siRNA solution | | |
| --- | --- | --- | --- | --- | --- | --- | --- | --- | --- |
|  |  |  |  |  |  |  |  |  |  |
|  |  |  | Flow rate  (μL/min) | Conc.  (μg/μL) | Vol.  (μL) |  | Flow rate  (μL/min) | Conc.  (μg/μL) | Vol.  (μL) |
| LSM1/PSM1 | 1:1 | 6 | 3 | 1.4 | 30 |  | 3 | 0.14 | 30 |
| LSM2/PSM2 | 5:1 | 6 | 5 | 0.84 | 50 |  | 1 | 0.42 | 10 |
| LSM3/PSM3 | 3:1 | 30 | 22.5 | 0.93 | 45 |  | 7.5 | 0.28 | 15 |
| LSM4/PSM4 | 1:1 | 54 | 27 | 1.4 | 30 |  | 27 | 0.14 | 30 |
| LSM5/PSM5 | 5:1 | 54 | 45 | 0.84 | 50 |  | 9 | 0.42 | 10 |

**Fig. S1.** The hydrodynamic diameter and polydispersity index (PDI) of LAH4-L1/siRNA complexes (LSM1-5) and PEG_12_KL4/siRNA complexes (PSM1-5) prepared with different total flow rates (TF) and flow rate ratio (FRR). The particle size distribution was measured at 20 to 360 min after mixing was completed (n=3). The smallest and the most stable complexes were obtained at TF of 6 µL/min and FRR of 1:1, and this condition was selected as the optimal protocol for the other evaluations.

**Table S2.** Factorial designs to optimise the microfluidic mixing method for the preparation of peptide/mRNA complexes. LMM refers to LAH4-L1/mRNA complexes; PMM refers to PEG_12_KL4/mRNA complexes. FRR is flow rate ratio (peptide to mRNA); TF is total flow rate.

|  | FRR | TF  (μL/min) | Inlet 1: peptide solution | | |  | Inlet 2: mRNA solution | | |
| --- | --- | --- | --- | --- | --- | --- | --- | --- | --- |
|  |  |  |  |  |  |  |  |  |  |
|  |  |  | Flow rate  (μL/min) | Conc.  (μg/μL) | Vol.  (μL) |  | Flow rate  (μL/min) | Conc.  (μg/μL) | Vol.  (μL) |
| LMM1/PMM1 | 1:1 | 6 | 3 | 1.4 | 30 |  | 3 | 0.14 | 30 |
| LMM2/PMM2 | 5:1 | 6 | 5 | 0.84 | 50 |  | 1 | 0.42 | 10 |
| LMM3/PMM3 | 3:1 | 18 | 13.5 | 0.93 | 45 |  | 4.5 | 0.28 | 15 |
| LMM4/PMM4 | 1:1 | 30 | 15 | 1.4 | 30 |  | 15 | 0.14 | 30 |
| LMM5/PMM5 | 5:1 | 30 | 25 | 0.84 | 50 |  | 5 | 0.42 | 10 |

**Fig. S2.** The hydrodynamic diameter and polydispersity index (PDI) of LAH4-L1/siRNA complexes (LMM1-5) and PEG_12_KL4/siRNA complexes (PMM1-5) prepared with different total flow rates (TF) and flow rate ratio (FRR). The particle size distribution was measured at 20 to 360 min after mixing was completed (n=3). The smallest and the most stable complexes were obtained at TF of 6 µL/min and FRR of 1:1, and this condition was selected as the optimal protocol for the other evaluations.

**Fig. S3A**. Transfection of LAH4-L1/siRNA complexes on A549 cells. Complexes were prepared at 10:1 ratio (w/w) by microfluidic or pipette mixing. siRNA targeting GAPDH (+) and negative control siRNA (-) were used. Cell transfected with Lipofectamine 2000 (Lipo2k)/siRNA (50 nM) and untreated cells (C) were used as controls. Western blot analysis of the GAPDH protein was performed at 48 post-transfection with β-actin used as internal control.

**Fig. S3B**. Transfection of LAH4-L1/siRNA complexes on BEAS-2B cells. Complexes were prepared at 10:1 ratio (w/w) by microfluidic or pipette mixing. siRNA targeting GAPDH (+) and negative control siRNA (-) were used. Cell transfected with Lipofectamine 2000 (Lipo2k)/siRNA (50 nM) and untreated cells (C) were used as controls. Western blot analysis of the GAPDH protein was performed at 48 post-transfection with β-actin used as internal control.

**Fig. S3C**. Transfection of PEG_12_KL4/siRNA complexes on A549 cells. Complexes were prepared at 10:1 ratio (w/w) by microfluidic or pipette mixing. siRNA targeting GAPDH (+) and negative control siRNA (-) were used. Cell transfected with Lipofectamine 2000 (Lipo2k)/siRNA (50 nM) and untreated cells (C) were used as controls. Western blot analysis of the GAPDH protein was performed at 48 post-transfection with β-actin used as internal control.

**Fig. S3D**. Transfection of PEG_12_KL4/siRNA complexes on BEAS-2B cells. Complexes were prepared at 10:1 ratio (w/w) by microfluidic or pipette mixing. siRNA targeting GAPDH (+) and negative control siRNA (-) were used. Cell transfected with Lipofectamine 2000 (Lipo2k)/siRNA (50 nM) and untreated cells (C) were used as controls. Western blot analysis of the GAPDH protein was performed at 48 post-transfection with β-actin used as internal control.

**Fig. S4A**. Transfection of LAH-4L1/siRNA complexes before and after nebulisation on A549 cells. Complexes were prepared at 10:1 ratio (w/w) by microfluidic mixing. siRNA targeting GAPDH (+) and negative control siRNA (-) were used. Western blot analysis of the GAPDH protein was performed at 48 post-transfection with β-actin used as internal control.

**Fig. S4B**. Transfection of LAH-4L1/siRNA complexes before and after nebulisation on BEAS-2B cells. Complexes were prepared at 10:1 ratio (w/w) by microfluidic mixing. siRNA targeting GAPDH (+) and negative control siRNA (-) were used. Western blot analysis of the GAPDH protein was performed at 48 post-transfection with β-actin used as internal control.

**Fig. S4C**. Transfection of PEG_12_KL4/siRNA complexes before and after nebulisation on A549 cells. Complexes were prepared at 10:1 ratio (w/w) by microfluidic mixing. siRNA targeting GAPDH (+) and negative control siRNA (-) were used. Western blot analysis of the GAPDH protein was performed at 48 post-transfection with β-actin used as internal control.

**Fig. S4D.** Transfection of PEG_12_KL4/siRNA complexes before and after nebulisation on BEAS-2B cells. Complexes were prepared at 10:1 ratio (w/w) by microfluidic mixing. siRNA targeting GAPDH (+) and negative control siRNA (-) were used. Western blot analysis of the GAPDH protein was performed at 48 post-transfection with β-actin used as internal control.
